# Zinc Homeostasis in Diabetes Mellitus and Vascular Complications

**DOI:** 10.3390/biomedicines10010139

**Published:** 2022-01-09

**Authors:** Stephanie MacKenzie, Andreas Bergdahl

**Affiliations:** Department of Health, Kinesiology and Applied Physiology, Concordia University, Montreal, QC H4B 1R6, Canada; stef.mackenzie@gmail.com

**Keywords:** oxidative stress, ROS, zinc homeostasis, zinc transporters

## Abstract

Oxidative stress represents an impaired metabolic system that promotes damage to cells and tissues. This is the predominant factor that leads to the development and progression of diabetes and diabetic complications. Research has indicated that zinc plays a consequential mechanistic role in the protection against oxidative stress as zinc is required for the proper functioning of the antioxidant system, the suppression of inflammatory mediators, and the modulation of zinc transporters. Recently, the mechanisms surrounding ZnT8, ZIP7, and metallothionein have shown to be of particular pathogenic importance and are considered as potential therapeutic targets in disease management. The literature has shown that zinc dysregulation is associated with diabetes and may be considered as a leading contributor to the deleterious vascular alterations exhibited by the disease. Although further investigation is required, studies have indicated the favorable use of zinc supplementation in the protection against and prevention of oxidative stress and its consequences over the course of the condition. This review aims to provide a comprehensive account of zinc homeostasis, the oxidative mechanisms governed by zinc status, current therapeutic targets, and the impact of zinc supplementation in the prevention of disease onset and in mitigating vascular complications.

## 1. Introduction

Diabetes mellitus (DM) is a metabolic disease defined as chronic hyperglycemia caused by insulin resistance (IR) or compromised insulin production [1]. These hyperglycemic conditions activate several pathways that generate reactive oxygen species (ROS), with the main source stemming from mitochondrial dysfunction and ER stress along with the polyol, hexosamine, protein kinase C (PKC), and advanced glycation end products (AGEs) pathways [2]. Consequently, the over-production of ROS (superoxide (O_2_^•−^), hydrogen peroxide (H_2_O_2_), hydroxyl radicals (⋅OH), and NADPH-oxidase (NOX)) coupled with reduced antioxidant capacity promotes a pathological imbalance that leads to oxidative stress and inflammation [2,3,4]. Stemming from these alterations, a wide array of diabetic complications is provoked including diabetic nephropathy, neuropathy and retinopathy, as well as the development of cardiovascular diseases (CVDs) [5,6]. In addition, individuals with diabetes exhibit a significantly increased risk of heart disease and stroke, and cardiovascular complications are the leading cause of morbidity and mortality within this population [7].

The divalent metal cation zinc (Zn^2+^) exhibits multiple physiological actions as a cofactor for over 300 enzymes and 2000 transporters [4,5,6,7,8]. As Zn^2+^ can be found in approximately 10% of human proteins, many types of physiological activity depend on Zn^2+^ homeostasis [8]. Zn^2+^ plays an important role in the antioxidant defense system that helps maintain a homeostatic balance by neutralizing ROS. Consequently, a Zn^2+^-deficient state reduces the buffering capability of the endogenous defense system in response the over-production of ROS exhibited by DM [9].

Zn^2+^ acts as an anti-inflammatory agent, provides structural stability to cell membranes, is an important regulator of gene expression, is needed for the correct functioning of glucose and lipid metabolism, mediates cell signaling pathways for cell proliferation and homeostasis, functions as an insulin mimetic and participates in the synthesis, storage, and secretion of insulin, inhibits pro-oxidant enzymes (nicotinamide adenine dinucleotide phosphate oxidase (NADPH-oxidase)), and is critical for the expression of superoxide dismutase (SOD), glutathione peroxidase (GPX), catalase, and metallothionein (MT), which are potent antioxidants that act as ROS scavengers [4,9,10,11,12]. Moreover, the recent literature has demonstrated an association between Zn^2+^ deficiency and diabetes: a distorted cycle where decreased Zn^2+^ bioavailability subsequently reduces the body’s ability to combat rising levels of pro-inflammatory, pro-apoptotic and pro-oxidant agents [13,14,15]. Fundamentally, the association between DM and the Zn^2+^ status is regulated by oxidative stress, inflammation, modulation of Zn^2+^ transporters, and impaired glucose and lipid metabolism [12].

**Figure 1:**
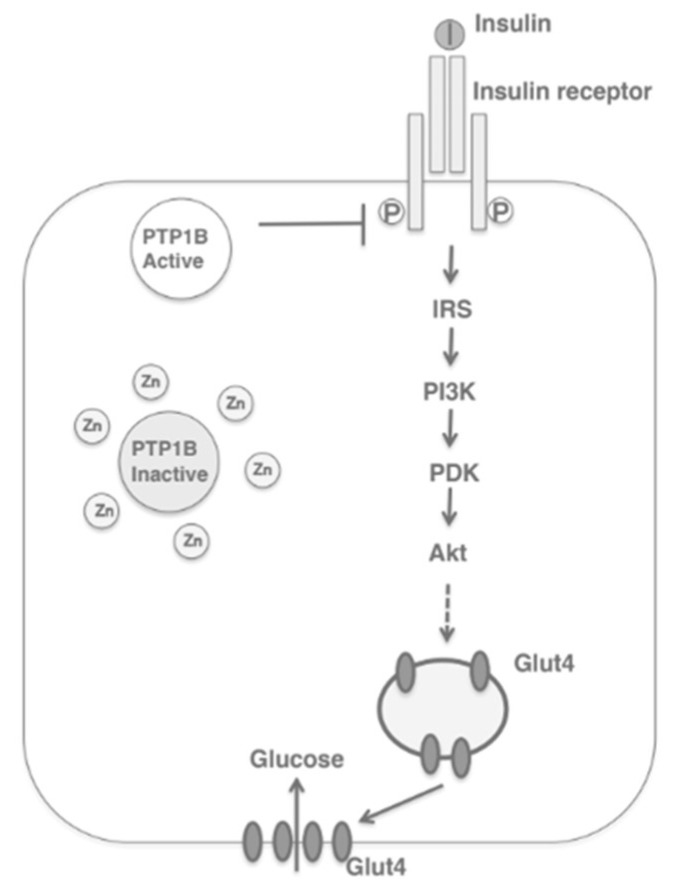
Zinc acting as an insulin-mimetic through its direct effect on the insulin-signaling pathway. IRS, insulin receptor substrate; PI3K, phosphatidylinosol-3-kinase; PKD, protein kinase D; Akt, protein kinase B [14].

Studies using diabetic mice or rats have observed that Zn^2+^ supplementation is cardioprotective and decreases oxidative stress as well as inflammatory markers [16,17,18]. Zn^2+^ supplementation in gestational diabetes has shown to reduce fasting plasma glucose and insulin levels [19]. And several studies in humans indicated that an inadequate intake of Zn^2+^ may increase the risk of diabetes onset [20,21,22]. As seen in Figure 1, this suggests that Zn^2+^ supplementation, particularly in Zn^2+^-deficient populations, may prevent disruptions of glucose homeostasis [14]. However, further investigation is required in order to ascertain the precise efficacy of Zn^2+^ supplementation.

Zn^2+^ homeostasis impacts both the pathophysiology of DM as well as the consequences of this metabolic disease. As such, the development of therapeutic solutions depends upon a better understanding of zinc’s properties and the extent to which Zn^2+^ can mediate diabetes-induced pathogenic changes and protect against cardiovascular complications. This review will examine the functional role of Zn^2+^ and its transporters, the association of the Zn^2+^ status with inflammation, oxidative stress, DM, and the possible mechanisms surrounding zinc’s protective role following supplementation.

## 2. Zinc Homeostasis

As Zn^2+^ plays a diverse role in cellular processes including cell signaling, enzymatic activity, and gene transcription, homeostatic mechanisms are required to tightly control Zn^2+^ absorption, distribution, intracellular availability, and excretion [15,23]. The cation Zn^2+^ cannot cross lipid bilayers and consequently physiological levels are maintained by three groups of proteins which regulate the inflow, outflow, and compartmentalization of Zn^2+^: the ZnT and ZIP families of Zn^2+^ transporters and the Zn^2+^-sensitive metallothioneins (MTs) [9,15,23,24]. The ZnT family (SLC30A) is a group of 10 (ZnT1-ZnT10) cation-diffusion facilitators that transport Zn^2+^ ions towards the extracellular space or from the cytosol into organelles [10,14]. The ZIP family (SLC39A), ZIP1-ZIP14, passes Zn^2+^ into the cytoplasm from the extracellular space or from intracellular organelles [10,11,14]. Zn^2+^ binds with MTs until homeostatic conditions change such that Zn^2+^ is required to be released and redistributed in the cells (e.g., in a state of oxidative stress, Zn^2+^ is released from its complex with MT for antioxidant purposes) [4,15].

Movement of the cation is facilitated within a bimodal framework of Zn^2+^ signaling. Early zinc signaling (EZS) is independent of gene transcription and results in a rapid fluctuation of intracellular Zn^2+^ levels via efflux from the organelles into the cytosol [11]. Late zinc signaling (LZS) is slower than the response of EZS because it consists of transcriptional changes in genes and includes the use of storage proteins or transporters. Together, both systems regulate processes involved in metabolism, cell differentiation, proliferation, and growth [11,12,13,14,15]. In the liver or muscle, evidence of Zn^2+^ on cellular signaling is exemplified through the inhibition of protein tyrosine phosphatase 1B (PTP1B). This protein negatively regulates insulin-signaling pathways whereas Zn^2+^ can extend the insulin signal through the insulin receptor via the inhibition of PTP1B [10,11]. The human MT family consists of 12 operational MTs, with MT1 and MT2 as the major isoforms in the pancreas; these molecules bind Zn^2+^ with high affinity, incorporating up to seven Zn^2+^ ions per molecule [8,9,25,26]. As MTs are equipped with reversible dissociation, they can act as Zn^2+^ donors or acceptors [8,9,25,26]. Downstream cell signaling is induced by releasing Zn^2+^ either via the oxidation of the sulfur donors in the MT molecule or through the interaction of the MT with nitric oxide (NO) [25].

### Zinc Distribution in the β-Cell

To understand the molecular pathways involved in DM and disease processes like IR it is necessary to uncover the contribution that Zn^2+^ and Zn^2+^-transporter mechanisms make towards cell signaling. This will provide the comprehension necessary to delineate the important molecules or pathways that have the greatest therapeutic potential [11].

Generally, mammalian cells cannot withstand high concentrations of Zn^2+^ as it can quickly cause toxicity; however, a small amount is necessary to maintain homeostatic functioning, hence the classification of Zn^2+^ as an essential micronutrient. Unlike elsewhere in the body, a healthy pancreas contains relatively high levels of the mineral [9]. The predominant source of pancreatic Zn^2+^ is found within the β-cells; specifically, they are contained in the dense cores of insulin secretory granules (ISGs). This presence highlights the critical nature of Zn^2+^ for insulin processing and storage [9,14].

**Figure 2:**
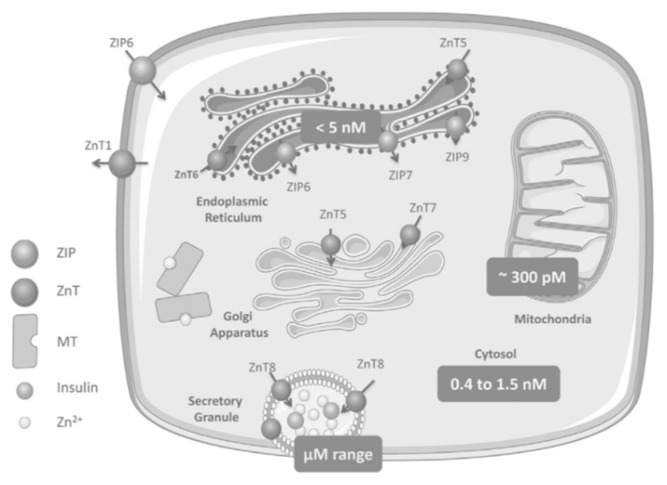
Zinc homeostasis in pancreatic beta cells includes multiple ZnT and ZIP transporters with ZnT8 transporters specific to the ISGs of pancreatic cells [9].

As shown in Figure 2, ZIP6 transports Zn^2+^ from the extracellular area into the β-cell, while ZnT1 moves Zn^2+^ from the cytosol towards the extracellular space. ZnT5 and ZnT6 move Zn^2+^ from the cytosol into the endoplasmic reticulum (ER), while ZIP6, ZIP7, and ZIP9 perform the reverse. ZnT5 and ZnT7 transport Zn^2+^ from cytosol to the Golgi apparatus and ZIP7, ZIP9, ZIP11, and ZIP13 work moving Zn^2+^ out of the Golgi into the cytosol (not pictured) [11].

ZnT8 is expressed with high specificity to pancreatic cells; it is a transmembrane protein of the ISG in islet beta and alpha cells and moves Zn^2+^ from the cytosol into these cells [27]. The selective site and function of ZnT8 establishes its immediate role in glucose homeostasis and insulin biology.

Within the β-cell, an inactive, single-chain preproinsulin molecule is produced in the rough endoplasmic reticulum (ER) and is subsequently cleaved to form proinsulin [28]. Transporter ZnT8 imports this into the ISG where maturation occurs followed by a second cleavage generating a c-peptide along with a native insulin molecule [9]. With adequate levels of Zn^2+^ and insulin, a hexamer is formed, and to obtain maximum storage capacity within the secretory vesicle, the hexamerization process decreases insulin solubility and produces crystallized proinsulin [9,28]. When blood glucose levels are high, the hexamers are converted into active monomers and expelled into the extracellular medium while concurrently freeing up a considerable concentration of Zn^2+^. Implications of whether these ions act downstream to further manage the actions of insulin or proceed to other tasks independent of insulin are unclear [9].

## 3. Zinc Transporters: Glucose Homeostasis, Insulin Resistance and Immunity

### 3.1. ZnT8

Due to the specificity and purpose of ZnT8 within the pancreatic β-cells, the recent literature has examined the potential therapeutic and diagnostic roles of this transporter. Huang et al. (2019) reviewed the functions of ZnT8 in diabetes, highlighting common gene polymorphisms and mutations that may increase the risk of Type 2 diabetes mellitus (T2DM) or have protective effects, respectively. Carriers of the SLC30A8 risk allele have lower insulin secretion, less conversion of proinsulin to insulin, decreased insulin sensitivity, and attenuated β-cell function. Meanwhile, other SLC30A8 mutations were linked with the therapeutic efficacy of antidiabetic drugs. The review by Nourazi et al. (2017) shared similar evidence where gene polymorphisms of ZnT8 and carriers of certain risk alleles played a part in the pathogenesis and likelihood of developing T2DM. Fukunaka and Fujitani (2018) suggested that ZnT8 levels determine the risk of T2DM development in mouse models. Electron microscopy analyzed the presence of dense ISGs in β-cells and found that ZnT8-KO mice presented irregular granules with distorted or empty cores. These mice also displayed mild glucose intolerance compared with the control group. The authors performed a pancreas perfusion experiment which indicated that insulin secretion was enhanced in ZnT8-KO mice, but the majority of the insulin produced was degraded in the liver. This indicates that the ZnT8 transporter may play a role regulating insulin clearance in the liver. The authors concluded that ZnT8 is a critical actor in insulin delivery to peripheral organs and subsequently on overall glucose metabolism.

Meanwhile, other studies in the literature investigated the relationship between the ZnT8 transporter and the incidence of Type 1 diabetes mellitus (T1DM). Of note, Williams and Long (2019) examined the capacity of ZnT8 autoantibodies (ZnT8As) to predict T1DM through their use as biomarkers to reflect insulin secretory capacity in diabetic patients. They found that the accurate measurement of ZnT8A is an efficient method to identify those who are at risk of this disease. However, the review found contrasting evidence as to whether ZnT8A could effectively act as a biomarker for therapeutic effect.

In addition to these contributions, further studies in the literature focused on the mechanism of ZnT8 in the pathogenesis of DM. Specifically, in a review by Myers et al. (2012), ZnT8 was found to play a role in the pathogenesis of both T1 and T2 diabetes. It was found that ZnT8A targeted ZnT8 in 60%-80% of new onset instances in T1 patients. The β-cell dysfunction and cytotoxicity associated with T1DM has been linked to these autoantibodies [25]. In T2DM, ZnT8 overexpression was linked with increased glucose-stimulated insulin secretion versus down-regulation, which was consistent with reduced insulin secretion under hyperglycemic conditions. Furthermore, ZnT8 knock-out (KO) mice experienced problems with glucose intolerance and ZnT8-null mice had diet-dependent issues of glucose tolerance, insulin secretion, and body weight. Adulcikas et al. (2019) demonstrated comparable results in ZnT8 null mice: decreased insulin secretion, compromised glucose tolerance, and impaired β-cell function.

Based on previous research, it is clear that ZnT8 is a key player in insulin biology and by extension, glucose homeostasis, making it a prime target for diabetic therapies. Yet, the literature above underscores the complexity of this task. It is possible that a more coherent picture could be obtained by pairing the ZnT8 information with the emerging knowledge of other Zn^2+^ transporters.

### 3.2. ZIP7

While T2DM indicates major metabolic dysfunction and aberrant blood glucose levels, IR is the precursor to this disease and is marked by the body’s inability to respond properly or in a timely fashion [10]. IR is induced by the distortion of accurate cell signaling and effective glucose uptake into peripheral tissues [11]. ZIP7 is responsible for transporting Zn^2+^ out of the ER and Golgi into the cytosol; it also controls cell signaling pathways (of note, the insulin receptor substrate-phosphoinositide-3-kinase-protein kinase B (IRS-P13K-AKT) pathway) akin to insulin, which initiates glucose uptake in skeletal muscle [10,11]. Literature suggests that ZIP7, as linked with ER stress and cell-signaling pathways, is at the root of IR-associated distortions.

**Figure 3:**
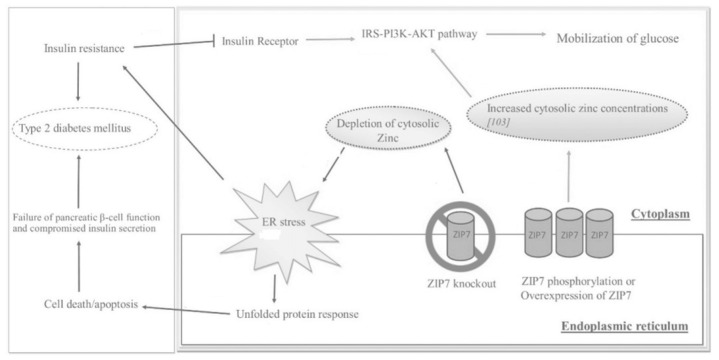
The effects of ZIP7 on glucose mobilization, ER stress, IR, and T2DM as depicted through various regulatory pathways. Adapted from [10].

As seen in Figure 3, ZIP7 action increases the cytosolic Zn^2+^ concentration, which participates in a signaling pathway that elicits glucose mobilization and metabolism [10,29]. In this regard, Zn^2+^ acts as an insulin mimetic through the phosphorylation of AKT and the resultant mobilization of Glut4 transporters to facilitate the influx of glucose in skeletal muscle [11]. In a Zn^2+^-depleted cytosolic environment, ER protein folding becomes compromised, thereby activating the unfolded protein response (UPR); the rate of folding is thus reduced, but if ER stress does not resolve, apoptosis is triggered [10].

When tested, the ablation of the transporter led to a significant reduction in cytosolic Zn^2+^ levels, an increased ER zinc content, cell proliferation abnormalities, and ER stress [23]. These findings were congruent with the research done by Anzilotti et al. (2019), where mass spectrometry that was performed on cells exhibiting the loss of ZIP7 displayed low concentrations of cytoplasmic Zn^2+^ and increased Zn^2+^ concentrations within the ER. As a result of Zn^2+^ depletion, ER stress can enhance ROS production and subsequent oxidative stress while byproducts of ER stress are also known to activate inflammatory pathways leading to endothelial dysfunction [2]. Therefore, a normal Zn^2+^ status and the ensuing ZIP7 expression is crucial to curtail ROS generation, impaired glucose uptake, β-cell failure, and IR [10,11].

Prior research demonstrated that low ZIP7 levels in skeletal muscle cause a significant reduction in Glut4 expression, in the phosphorylation of AKT, and defective glycogen synthesis [30]. Other studies indicated that hyperglycemic conditions caused the up-regulation of ZIP7 in order to alleviate ER stress in the pancreatic islet β-cells of mice and in rat cardiomyocytes [31,32]. The association between ZIP7 and the pathogenesis of DM was further examined by Norouzi et al. (2019) in insulin-resistant skeletal muscle and mice that were fed a high-fat diet. IR was induced by the treatment of skeletal muscle with an insulin receptor inhibitor or palmitate, and mice were fed either a high-fat diet or normal chow for 10 weeks. Immunoblotting conducted on increasing concentrations of glucose in normal skeletal-muscle cells confirmed that glucose up-regulates ZIP7 expression. However, the expression of ZIP7 and Glut4 were suppressed in the insulin-resistant cells. Furthermore, the high-fat-diet mice exhibited a significant reduction of ZIP7 and Glut4 expression compared to the control mice when skeletal-muscle tissue was analyzed from both cohorts.

While current literature suggests that ZIP7 may be a contributing factor in the pathogenesis of T2DM, the Zn^2+^-mediated mechanisms that are involved in skeletal-muscle glycemic control require further investigation, particularly via in vivo studies of mice and humans.

### 3.3. Metallotionein

MT is a potent ROS scavenger that offers significant protection against DM and DM-induced cardiovascular injury [33]. Studies of Zn^2+^ supplementation in diabetic mice have shown that the expression of MT is significantly induced by cellular Zn^2+^ levels [17,33]. This mechanism has been further confirmed as Zn^2+^ supplementation up-regulates the expression of MT and consequently decreases diabetes-induced vascular complications [17]. Research has shown that MT single nucleotide polymorphisms are related to various pathological processes, three of which are linked with a significant increase in T2DM prevalence due to reduced MT antioxidant capabilities [8]. Zn^2+^ effectively determines MT levels through the stimulation of responsive metal transcription factor 1 (MTF-1); this transcription factor directly regulates the expression of MT [2].

A review by Choi et al. (2018) described the importance of MT’s antioxidant capabilities whereby reduced MT activity contributes to the pathology of DM [25]. These decreased concentrations were linked with an increased susceptibility to hyperglycemia and oxidative stress in T2DM. Concurrently, the overexpression of MT in streptozotocin-induced diabetic mice caused lower levels of β-cell DNA damage and oxidative stress. While this classical view of MT as an antioxidant has been confirmed, a review by Park et al. (2018) suggests new mechanisms of action for MT [34].

First, the authors proposed that MT may counteract oxidative stress by reversing mitochondrial dysfunction, which is the main source of ROS. Mitochondrial dysfunction is a primary contributor to many diabetic issues including β-cell impairment, IR, endothelial dysfunction, and vascular damage [6,34]. The precise mechanism surrounding MT’s effects on the respiratory chain are unclear, but studies have demonstrated that MT protects against mitochondrial superoxide (O_2_^•−^) over-production and exerts myocardial anti-apoptotic effects induced by mitochondrial dysfunction [34,35,36]. Other promising diabetic MT protective mechanisms suggested by Park et al. (2018) include: the mitigation of oxidative-induced ER stress via the suppression of signaling pathways, the maintenance of cellular autophagy to prevent apoptosis, and the possible anti-inflammatory action of extracellular MTs in the immune system and neuronal health.

Interestingly, Choi et al. (2018), suggested the use of MT in the treatment of DM and DM-induced CVDs. As Zn^2+^ homeostasis plays a prominent role in disease pathology and diabetic complications, an accurate method to analyze Zn^2+^ levels is required. The recent literature has proposed the use of Zn^2+^ transporters or Zn^2+^-binding proteins (MTs) in circulating blood cells as biomarkers of the Zn^2+^ status. As Zn^2+^ itself is not a reliable indicator, the expression of MT in leukocytes appears to be the favorable option due to the rapid and dose-dependent manner in which MT responds to changing Zn^2+^ levels. In addition, Zn^2+^ transporters and MTs may also hold diagnostic and prognostic abilities for CVDs. The authors described how emerging research on extracellular vesicles (EVs) from human liquid biopsies could act as disease markers, in this case, specifically EVs from endothelial or cardiac tissue. Further investigation to link EVs with a specific Zn^2+^ transporter or MT, and CVDs will be required in order to uncover the true diagnostic and prognostic value of the Zn^2+^ status. However, studies have shown that stress-induced levels of MT have been detected in endothelial exosomes, and Zn^2+^ transporters were present among other samples of EVs.

### 3.4. Other ZnT/SLC30A Transporters

The efflux of Zn^2+^ in smooth muscle cells is regulated by the ZnT1, ZnT5, and ZnT9 transporters [25]. Studies have shown that levels of ZnT1 and ZnT2 are impacted by dietary intake of Zn^2+^ while ZnT4 expression is not diet-dependent [15]. ZnT3 transporters are predominantly located within neurons, transporting Zn^2+^ to the synaptic vesicles of glutaminergic hippocampal neurons [8,14]. ZnT3-null mice have been shown to demonstrate reduced insulin gene expression and secretion [15]. The overexpression of ZnT5 and ZnT7 can prevent cellular death in hyperglycemic conditions, whereas the inhibition of these transporters is linked to an increase in apoptosis [8]. ZnT5 was also found to be abundantly expressed in human endothelial cells while ZnT9 was highly expressed in human and rat hearts [25]. Some studies have suggested that ZnT7 plays a redundant role of ZnT8 in the pancreas but this remains unclear as ZnT7-KO and ZnT8-KO studies have illustrated inconclusive results [8,14].

### 3.5. Other ZIP/SLC39A Transporters

The ZIP1 transporter, regulated by testosterone and prolactin, is linked with rapid cellular accumulation and uptake of Zn^2+^ in cells [15]. ZIP1 and ZIP13 are also the dominant SLC39A transporters expressed in human endothelial tissue [25]. ZIP6 impacts insulin secretion in pancreatic β-cells, whereby the down-regulation of the transporter results in dysfunctional insulin secretion in response to glucose [16]. ZIP8 action increases intracellular Zn^2+^ levels and may also play a role in lung epithelial cells [15]. Zn^2+^ that is passed to macrophages and monocytes by ZIP8 under inflammatory conditions highlights its key role in protecting against inflammation [12]. ZIP13 is associated with beige adipocyte synthesis and energy metabolism through the use of Zn^2+^ to inhibit adipocyte browning [37]. It was found that ZIP13-KO mice produced higher levels of beige adipocytes and consequently had an improved glucose metabolism and insulin tolerance [14]. ZIP14 can transport Zn^2+^, iron, and manganese, with up-regulation occurring under pro-inflammatory conditions (stress, acute infection, inflammation) when there are elevated concentrations of interleukin-6 (IL-6) and NO [15,38]. The acute phase response during inflammation will up-regulate ZIP14 for the rapid intake of plasma Zn^2+^ into the organs, primarily the liver, to limit Zn^2+^ availability for invading pathogens [39]. Additionally, it has been noted that ZIP1, ZIP7, ZIP13, and ZIP14 are highly expressed in human heart tissue [25]. A summary of zinc transporters, regulators, and effects can be found in Table 1.

## 4. Zinc, Inflammation, Oxidative Stress and Vascular Complications

Zn^2+^ deficiency, a risk factor for DM, is closely associated with increased levels of oxidative stress and the generation of inflammation [12]. A vicious cycle between inflammation and oxidative stress is crafted through pro-inflammatory transcription factors (e.g., nuclear factor kappa-light-chain-enhancer of activated B cells: NF-kB) inducing ROS production and inflammatory cytokine release (IL-6, etc.), which in turn intensifies oxidative stress [4]. The NF-kB signaling pathway regulates the expression of pro-inflammatory cytokines, acute phase proteins (C-reactive protein: CRP), matrix metalloproteinases (MMPs), and is responsible for genes that dictate apoptosis, proliferation, cell adhesion, tissue remodeling, cellular-stress responses, inflammatory processes, and immune responses [12,39]. Within this framework, the accumulation of vascular oxidative stress results in endothelial dysfunction whereby cells activate an inflammatory response that further perpetuates the cycle [7,40]. These circumstances lead to an increase in apoptotic cells, extracellular matrix remodeling, and further endothelial dysfunction [40]. Dysfunction at the cellular level triggers vascular dysfunction which ultimately results in both micro- and macro-vascular diseases [1,7,40].

**Figure 4:**
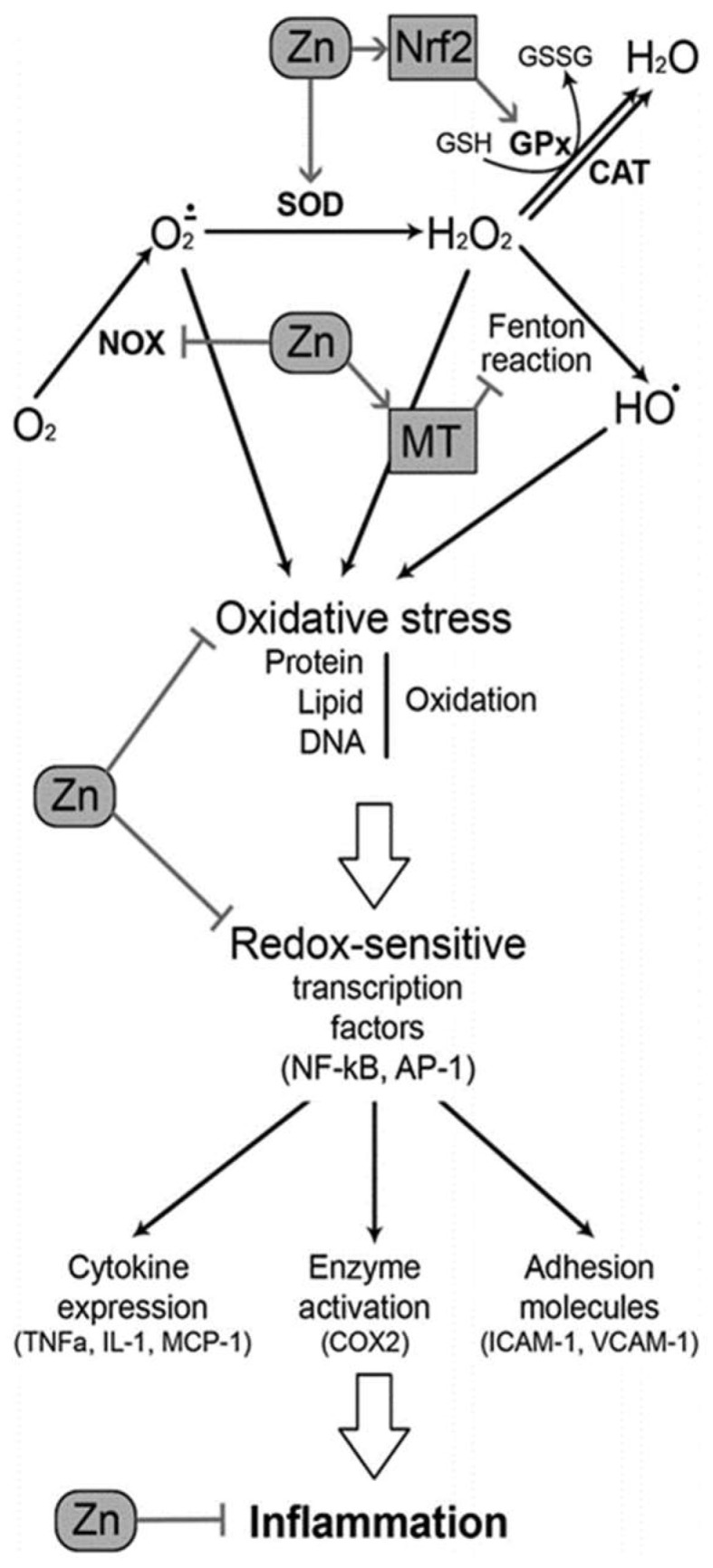
The various pathways in which zinc influences oxidative stress and inflammation. Adapted from [12].

As seen in Figure 4, the presence or absence of Zn^2+^ regulates NF-kB transcription. Zn^2+^ deficiency induces the activation of NF-kB, promoting oxidative stress, whereas Zn^2+^ homeostasis alleviates inflammation and oxidative damage [39]. This is accomplished via the anti-inflammatory, anti-apoptotic, Zn^2+^-protein complex A20 and the peroxisome proliferator-activated receptors (PPARs) [4,12,39]. A study of endothelial cells demonstrated that cells with high Zn^2+^ content had an up-regulated expression of A20 which decreased NF-kB activity and thus suppressed the production of pro-inflammatory cytokines [41]. Moreover, Zn^2+^ supplementation was shown to increase the concentration of A20 and PPAR-α, thereby inhibiting NF-kB signaling and thus reducing the levels of ROS, inflammatory cytokines, and adhesion molecules that would otherwise lead to the development of atherosclerosis [42]. Another mechanism of Zn^2+^-induced NF-kB inhibition is accomplished via Zn^2+^ ion transportation through the up-regulation of ZIP8 into macrophages and monocytes during inflammatory conditions [12]. These Zn^2+^ ions block the downstream activity of IkB kinase complex (IKK) and ergo is unable to phosphorylate and degrade IkB, which is the inhibitor of NF-kB; ultimately, this prevents the translocation of NF-kB and its ability to target gene expression [39]. Zn^2+^ is also a known inhibitor of N-methyl-D-aspartate (NMDA); a Zn^2+^ deficiency activates this receptor to produce elevated levels of intracellular calcium which, in turn, promotes neuronal cells to release substance P, causing increased concentrations of inflammatory cytokines, free radicals, and oxidative stress [4]. Additionally, the Zn^2+^ status contributes to inflammation and atherosclerosis by its regulation of Zn^2+^-dependent MMPs, where inadequate levels of Zn^2+^ initiate pathological signaling pathways [12].

Furthermore, oxidative stress can impair protein folding in the ER, and conversely, improper protein folding brought on by ER stress (as a result of Zn^2+^ depletion) can enhance ROS production [2]. This highlights the critical nature of Zn^2+^ homeostasis such that the ER Zn^2+^ transporter ZIP7 is properly expressed to mitigate ROS generation, impaired glucose uptake, β-cell failure, and IR [10,11]. The induction of ER-stress byproducts are also known to participate in the activation of an NF-kB pathway that facilitates inflammation, endothelial dysfunction, and the progression of diabetic complications [2].

**Figure 5:**
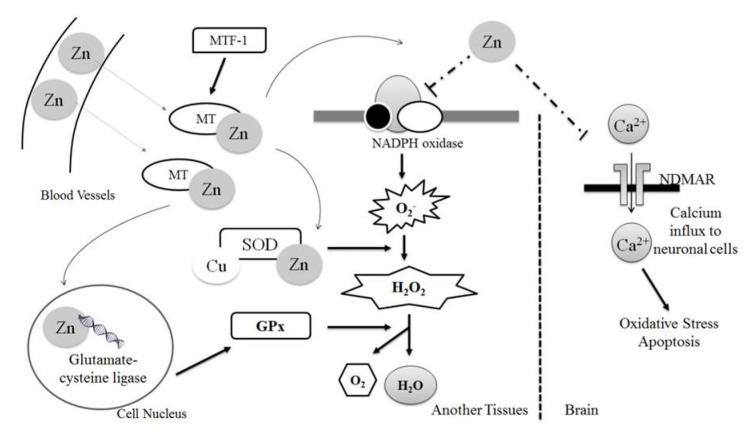
The mechanistic role of zinc in the antioxidant defense system [4].

In the endogenous defense system, depicted in Figure 5, Zn^2+^ has the capacity to influence antioxidant functioning through direct and indirect methods. Zn^2+^ directly inhibits the pro-oxidant enzyme NADPH-oxidase and also contributes to the proper functioning of many antioxidant ROS scavengers; the metal has a strong influence on the expression of MT, it is a structural component of SOD, and it promotes the expression of an enzyme (glutamate cysteine ligase) involved in GPX synthesis [4]. The transcription factor, nuclear factor erythroid-2-related-factor 2 (Nrf2) controls the expression of the genes that encode these antioxidants and others, and it is Zn^2+^ that regulates Nrf2 activity [39]. Studies of Zn^2+^-deficient mice have shown a significant reduction in Nrf2 expression in conjunction with elevated levels of oxidative damage [43].

As a result, Zn^2+^ deficiency provokes an environment where the body is unable to defend against an accumulation of oxidative damage. This oxidative stress induces endothelial dysfunction and atypical changes in vascular smooth muscle cells, promoting the propagation and migration of these cells in atherosclerotic lesions [2]. However, a healthy Zn^2+^ status and the regulation of Nrf2 mitigate the damage done to cells and vessels per the expression of antioxidants while buffering the ROS generated from hyperglycemia, inflammation, and ER stress [4].

It is clear that Zn^2+^ deficiency in DM promotes the rampant expression of immune mediators while enhancing oxidative stress and an immune response that is unable to be constrained; the outcome leads to many pathological changes that aggravate disease progression and cardiovascular complications [12].

## 5. Zinc Supplementation

Emerging evidence on the complex yet critical nature of Zn^2+^ homeostasis in the pathophysiology of diabetes has shifted research focus towards the analysis of therapeutic Zn^2+^ supplementation. Zinc deficiency can result from a number of factors: (i) inadequate intake or absorption, (ii) loss of zinc, mainly through urine and (iii) increased daily needs.

A large portion of previous research has focused on the use of Zn^2+^ supplementation in the form of ZnSO_4_ relative to diabetic cardiomyopathy. Lu et al. (2015) used diabetic rats to assess the effects of Zn^2+^ supplementation on cardiac tissue [44]. Their results demonstrated a partial protection against diabetic cardiomyopathy through decreased oxidative stress and autophagy in cardiac tissue. Wang et al. (2006) also investigated the consequences of intraperitoneal zinc sulfate supplementation against diabetic cardiomyopathy in a murine model but through a mechanism driven by MT. They found an increase in cardiac MT due to Zn^2+^ binding and their results indicated that Zn^2+^ supplementation protected cardiac cells against free-fatty-acid cytotoxicity. Another study in db/db mice by Wang et al. (2017) looked at the role of various Zn^2+^ levels (deficient, adequate, or supplemented through diet) in diabetic cardiomyopathy development and progression. The mice that were fed a Zn^2+^-deficient diet had increased non-fasting blood glucose levels and intraperitoneal glucose intolerance, decreased levels of hepatic Zn^2+^ and ejection-fraction percentage, and increased cardiac fibrosis and left-ventricle mass. The group receiving adequate Zn^2+^ levels did not experience these effects and the cohort taking additional Zn^2+^ supplementation had no significant changes when compared to the adequate Zn^2+^ diet. Zn^2+^-deficient mice also had an increased expression of inflammatory markers while the Zn^2+^-supplemented diet alleviated this compared to the adequate Zn^2+^ diet. It was shown by Cooper-Capetini et al. (2017) that ZnCl_2_ supplementation in water for mice that were fed a high-fat diet promoted pancreatic β-cell function as seen through a revived glucose-stimulated insulin secretion, glucose tolerance, and improved HOMA-β [45]. Additionally, Zn^2+^ supplementation was found to up-regulate MTs in MDCK cells and subsequently to alleviate oxidative stress and apoptosis [19]. T1-diabetic mice treated with ZnSO_4_ experienced a significant reduction in hepatic oxidative stress, ER stress, and cell death compared to the controls [46]. In addition, the progression of diabetic nephropathy in rats was relieved by zinc carbonate supplementation by reducing the overexpression of molecular markers associated with oxidative stress [47].

In humans, a double-blind, randomized control trial was conducted with 200 pre-diabetic subjects in order to assess the effects of Zn^2+^ supplementation over a year [20]. The Zn^2+^ group received 20 mg of Zn^2+^ daily, while the control group was given a placebo. Significant decreases were found in fasting plasma glucose, the 2 h oral glucose-tolerance test, and plasma glucose in the Zn^2+^ group compared to their controls. Of the control group, 25% went on to develop diabetes versus 11% in the Zn^2+^ group; however, the differences in significance levels for disease progression were unclear. Zn^2+^ supplementation was also studied in women with gestational diabetes by reducing the subjects’ serum insulin, insulin resistance, and fasting plasma glucose [12]. Furthermore, a meta-analysis of five randomized controlled trials with 263 subjects that examined Zn^2+^ supplementation in gestational diabetes concluded that although LDL and total cholesterol were unaffected, the treatment decreased measurements of insulin, fasting plasma glucose, and HOMA-IR [19]. In the United States, a prospective cohort study examined 82,000 women and found that the insufficient intake of dietary Zn^2+^ caused a 17% increased risk of developing diabetes versus women with acceptable amounts of Zn^2+^ [14,22]. However, these results need to be read with caution as zinc from food sources is less bioavailable than supplements, as the former biologically competes with other minerals such as iron. A study in China suggested that glucose tolerance and the risk of diabetes is controlled by the interaction of ZnT8 dysfunction and the reduced levels of plasma Zn^2+^ [21]. However, in other cases, the link between the Zn^2+^ status, glucose metabolism, and IR lacked clarity [12]. While most of these results indicate that Zn^2+^ supplementation, particularly in Zn^2+^-deficient populations, may prevent disruptions of glucose homeostasis, the efficacy of such supplementation remains inconclusive and requires further investigation [14]. Zn^2+^ has multiple essential functions and could interact with many biological mechanisms to induce adverse effects. Very high concentrations of Zn^2+^ may have deleterious effects and may cause nausea, abdominal cramping, vomiting as well as an undesirable increase in blood pressure [14]. In addition, having a disproportionally high intake of oral zinc relative to copper could lead to copper deficiency. The effects include decreases in copper-dependent enzymes such as superoxide dismutase and cytochrome c oxidase. Consequently, in dietary and supplemental intakes, zinc and copper should be proportionate [12].

## 6. Conclusions

The full extent of zinc’s role in the molecular mechanisms involved in the pathogenesis and pathophysiology of many chronic diseases, including diabetes, has not yet been fully uncovered [4]. Yet, the current literature acknowledges the deleterious effects of dysregulated Zn^2+^ homeostasis in diabetes and its role in the development of both micro-vascular and macro-vascular complications [2,4,25,39]. Inadequate levels of Zn^2+^ result in impaired antioxidant functioning, cytokine over-expression, chronic inflammation, ROS accumulation and oxidative damage, distorted lipid and glucose metabolism, ER stress, β-cell defects and apoptosis, IR, endothelial dysfunction, and the evolution of cardiovascular complications [12]. Zn^2+^ depletion is now a hallmark characteristic of DM and is at the core of many pathogenic changes driven by hyperglycemia-induced oxidative stress and DNA damage. As such, the clinical management of Zn^2+^ nutrition over the course of the condition is paramount in maintaining Zn^2+^ homeostasis and the subsequent regulation of MT and Zn^2+^-transporter expression [48].

Future research is required for the expression of Zn^2+^ transporters and MT as biomarkers for the Zn^2+^ status, along with additional analyses of EVs from liquid biopsies [25]. More prospective cohort studies are needed in order to ascertain whether Zn^2+^ supplementation is indeed an effective agent in the prevention of diabetes onset [14]. And further investigation of Zn^2+^ supplementation in humans is necessary such that a clinical standard of care, relative to Zn^2+^ therapy, may be implemented jointly with other treatments.

Although the complete mechanistic relationship between the Zn^2+^ status and the occurrence of chronic disease remains obscured, studies have established that Zn^2+^ deficiency, by the induction of inflammation and oxidative stress, promotes the onset/progression of many conditions (DM, metabolic syndrome, obesity, cancer, kidney disease, neurodegeneration, atherosclerosis, CVDs) [4,14,39]. The comprehensive effect of Zn^2+^ homeostasis on the immune system is undeniable; this suggests that restoring normal levels of the metal through supplementation may prove to be an effective therapeutic measure, not only for diabetes, but for overall human health.

## Figures and Tables

**Table 1:** Zinc transporters, regulators, and effect his is a table.

Zinc Transporter	Regulators	Effect
ZnT1	Metal-responsive mode of regulation; dietary intake of zinc	Efflux of zinc in smooth muscle cells
ZnT2	Metal-responsive mode of regulation; dietary intake of zinc	Zinc transport in vesicles and lysosomes of pancreas, kidney, testis, epithelial cells, small intestine
ZnT3	Glucose status	Transport of zinc to synaptic vesicles
ZnT4	Unaffected by changes in dietary zinc uptake; regulated by extracellular zinc concentrations	Transport of zinc in the trans-Golgi network and in the cytoplasmic vesicular compartment
ZnT5	Glucose status; zinc-responsive elements	Transport of zinc into Golgi lumen for storage
ZnT7	Glucose status	Transport of zinc to Golgi apparatus in retina, liver, epithelial cells, small intestine; may play a redundant role of ZnT8
ZnT8	Glucose status	Regulation of zinc in the secretory vesicles of pancreatic β-cells
ZnT9	Expressed in low levels in response to dietary intake of zinc	Export of zinc out of myocytes; efflux of zinc in smooth muscle cells
ZIP1	Testosterone and prolactin	Uptake of zinc into cells
ZIP6	Estrogen stimulation, glucose status	Down-regulation leads to poor insulin secretion
ZIP7	Glucose status	Increases cytosolic zinc concentrations that participate in glucose mobilization and metabolism
ZIP8	Glucose status, TNF-∝ in lung epithelial cells	Increases intracellular zinc levels
ZIP13	Gene mutation leads to loss of function	Inhibition of adipocyte browning
ZIP14	Acute phase response during inflammation; IL-6	Rapid intake of plasma zinc into the organs

## Data Availability

Not applicable.
